# Mechanism Underlying the Bypass of Apurinic/Pyrimidinic Site Analogs by *Sulfolobus acidocaldarius* DNA Polymerase IV

**DOI:** 10.3390/ijms23052729

**Published:** 2022-03-01

**Authors:** Qin-Ying Huang, Dong Song, Wei-Wei Wang, Li Peng, Hai-Feng Chen, Xiang Xiao, Xi-Peng Liu

**Affiliations:** 1 State Key Laboratory of Microbial Metabolism, School of Life Sciences and Biotechnology, Shanghai Jiao Tong University, 800 Dong-Chuan Road, Shanghai 200240, China; hqy76668022@sjtu.edu.cn (Q.-Y.H.); dsongad@connect.ust.hk (D.S.); www1037554814@sjtu.edu.cn (W.-W.W.); pl_sky@126.com (L.P.); haifengchen@sjtu.edu.cn (H.-F.C.); 2 Shanghai Institute of Applied Physics, Chinese Academy of Sciences, No. 239 Zhangheng Road, Shanghai 201204, China; 3 Joint International Research Laboratory of Metabolic & Developmental Sciences (Ministry of Education), Shanghai Jiao Tong University, 800 Dong-Chuan Road, Shanghai 200240, China

**Keywords:** AP site analogs, translesion synthesis, Dbh, little finger domain, *Sulfolobus acidocaldarius*

## Abstract

The spontaneous depurination of genomic DNA occurs frequently and generates apurinic/pyrimidinic (AP) site damage that is mutagenic or lethal to cells. Error-prone DNA polymerases are specifically responsible for the translesion synthesis (TLS) of specific DNA damage, such as AP site damage, generally with relatively low fidelity. The Y-family DNA polymerases are the main error-prone DNA polymerases, and they employ three mechanisms to perform TLS, including template-skipping, dNTP-stabilized misalignment, and misincorporation-misalignment. The bypass mechanism of the dinB homolog (Dbh), an archaeal Y-family DNA polymerase from *Sulfolobus acidocaldarius*, is unclear and needs to be confirmed. In this study, we show that the Dbh primarily uses template skipping accompanied by dNTP-stabilized misalignment to bypass AP site analogs, and the incorporation of the first nucleotide across the AP site is the most difficult. Furthermore, based on the reported crystal structures, we confirmed that three conserved residues (Y249, R333, and I295) in the little finger (LF) domain and residue K78 in the palm subdomain of the catalytic core domain are very important for TLS. These results deepen our understanding of how archaeal Y-family DNA polymerases deal with intracellular AP site damage and provide a biochemical basis for elucidating the intracellular function of these polymerases.

## 1. Introduction

Each organism in the three kingdoms of bacteria, archaea, and eukaryotes possesses more than one DNA polymerase for genome replication and DNA damage repair. Replicative DNA polymerases interact with many other proteins to form a complex replisome to synthesize chromosomal DNA [1]. In addition, to replicative DNA polymerases, other kinds of DNA polymerases perform an error-free repair of the damaged DNA, translesion synthesis (TLS), and somatic hypermutation of immunoglobulin genes [2]. Based on the sequence similarity and enzymatic properties, DNA polymerases are classified into six main groups: families A, B, C, D, X, and Y [3]. Families B and C are eukaryotic and bacterial replicative DNA polymerases, respectively [2,3]. However, archaea from different phyla use B- and/or D-family DNA polymerases to replicate their genomes [4]. DNA polymerases from other families mainly function as DNA polymerases during various DNA repair processes [2,5], such as Y-family polymerases, which are involved in DNA repair and the somatic hypermutation of immunoglobulin genes [6]. TLS is a specific DNA repair affair for severe DNA damage and is carried out by specific DNA polymerases. Generally, TLS DNA polymerases lack a 3′ to 5′ proofreading activity and synthesize DNA in an error-prone manner. Both Y- and X-family DNA polymerases are error-prone, but only the former participates in the bypass of various damages [7,8]. In addition to Y-family DNA polymerases, the error-prone replicative DNA polymerase DnaE (belonging to the C-family) was also found to be able to bypass severely damaged DNA, such as AP sites, and might participate in TLS in vivo [9]. Based on the sequence similarity, Y-family DNA polymerases are divided into six groups: the DinB (pol IV) and UmuDC subfamilies (pol V) of prokaryotes, and the Rad30A (pol η), Rad30B (pol ι), DinB1 (pol κ), and Rev1 subfamilies of eukaryotes [8,10].

In order to elucidate the catalytic mechanism, high-resolution structures of apo-Dbh, binary and ternary Dbh-substrate complexes have been obtained for almost every family of DNA polymerases [11,12]. The Y-family DNA polymerases share a structure that comprises an N-terminal catalytic core domain and a C-terminal Y-family-specific domain that is known as the little finger (LF) domain or polymerase-associated domain (PAD) [13,14,15]. The catalytic core domain is similar to that of all the other families of DNA polymerases and is divided into three structural subdomains: finger, palm, and thumb [14]. The LF domain is unique to the Y-family of polymerases and has a greater sequence variability than the catalytic core domain [15]. The DNA substrate is bound between the thumb and LF subdomains of the catalytic domain [13]. The catalytic group of the thumb subdomain, assisted by Mg^2+^ and the LF domain, interacts with the DNA template base and the nucleotide to be incorporated during the polymerization reaction [8].

The Y-family DNA polymerases employ three mechanisms (Figure S1), including the template-skipping [16], the dNTP-stabilized misalignment [17], and the misincorporation-misalignment [17,18], to perform TLS. The dinB homolog (Dbh) from *Sulfolobus acidocaldarius* (*S. acidocaldarius*), an archaeal error-prone DNA polymerase IV, has also been extensively studied in vitro as a model to elucidate the TLS mechanism at DNA damage sites. It was found that the Dbh achieves processive DNA synthesis by skipping a template base and generating a single-base deletion [19]. The position of this skipped template base strongly influences the extension speed of the Dbh at the single-base deletion site [19]. When the jumping base is located three bases upstream of the nascent base pair (defined as the −3 position), the extension is the fastest. Located at the position two bases upstream (defined as the −2 position), the extension is five times slower than at the −3 position, and at the −1 position, it is at least 30 times slower than at the −3 position. In summary, the extension speed for single-base deletion is in the order of −3 > −2 >> −1 [19]. However, the TLS mechanism of Dpo4, a dinB homolog from *Sulfolobus solfataricus*, was inferred to be a template-skipping [16,20] rather than a dNTP-stabilized misalignment [17,18]. Because of the slow rate of the mismatch formation in the Dbh, the misincorporation–misalignment is not the main mechanism of the Dbh [21].

Template skipping and dNTP-stabilized misalignment models have been proposed to better explain the mechanism of TLS by the Dbh [19]. However, the molecular mechanism of TLS for the AP site analogs by the Dbh still needs to be further elucidated. Using various primer–template (PT) DNA substrates with different structures and AP site analogs, the molecular mechanism of TLS by the Dbh was fully investigated. The results confirm that the Dbh primarily uses template skipping accompanied by a dNTP-stabilized misalignment mechanism to bypass the AP site analogs, and the incorporation of the first nucleotide across the damage site is most difficult. Based on the reported crystal structure, we further confirmed that three conserved amino acid residues, Y249, R333, and I295, in the LF domain, and one residue, K78, in the palm subdomain of the catalytic core domain of Dbh, are very important to TLS, indicating the importance of the LF domain of the Dbh during the TLS of damaged DNA. These results deepen our understanding of the template skipping mechanism of the Dbh in TLS and provide a biochemical basis for understanding its intracellular function in addressing AP site damage.

## 2. Materials and Methods

### 2.1. Materials

The spacer phosphoramidites dSpacer (dS), C3, C6, C12, S9, and S18 were purchased from Glen Research (Sterling, VA, USA) or ChemGenes Company (Wilmington, MA, USA) and were used to introduce the specific spacer arm into an oligonucleotide by solid–phase synthesis. The *S. acidocaldarius* strain was a gift from Professor Albers (Molecular Biology of Archaea, Institute of Biology, University of Freiburg, Freiburg, Germany), and its genomic DNA was extracted by phenol–chloroform. The expression vector pDEST17 and expression host *Escherichia coli* Rosetta2 (DE3) pLysS were used throughout this study. KOD-plus DNA polymerase was purchased from Toyobo (Osaka, Japan). Nickel-nitrilotriacetic acid resin was purchased from Bio–Rad (Hercules, CA, USA). All other chemicals and reagents were of analytical grade.

### 2.2. Expression and Purification of DNA Polymerase Dbh

The gene encoding Dbh (saci_0554) was amplified by PCR from *S. acidocaldarius* genomic DNA and inserted into the expression vector, pDEST17, according to our previously described method [22]. Recombinant Dbh was expressed in Rosetta 2 (DE3) pLysS cells by induction with 0.5 mM IPTG for 3 h at 30 °C. Recombinant Dbh was purified from the induced bacteria via immobilized Ni^2+^ affinity chromatography according to a standard method. After verifying the purity of the eluate using 15% sodium dodecyl sulfate–polyacrylamide gel electrophoresis (SDS–PAGE), the preparations were dialyzed against a storage buffer consisting of 20 mM Tris-HCl (pH 8.0), 200 mM NaCl, and 50% glycerol and then stored in small aliquots at −20 °C.

Amino acid substitutions were introduced into Dbh with the QuikChange^®^ Site-Directed Mutagenesis Kit (Santa Clara, CA, USA) using KOD-plus DNA polymerase and the appropriate primers (Table S1). The nucleotide sequences were confirmed by DNA sequencing. The mutant Dbhs were prepared with the same protocol as that used for the wild-type polymerase.

### 2.3. Translesion Synthesis Assay

DNA polymerase activity and TLS assays were performed in a reaction buffer consisting of 20 mM Tris-Cl (pH 8.8), 10 mM (NH_4_)_2_SO_4_, 10 mM KCl, 5 mM MgCl_2_, 0.1% Triton-X100, 0.1 mg/mL BSA, and 0.2 mM dNTPs. The PT DNA substrates were prepared by annealing the 5′-6-FAM-labeled primer strand with the complementary template strand in a molar ratio of 1:1.5. The natural spacer deoxyribose (Figure S1A) is not thermostable and is not suitable for the biochemical characterization of the thermostable protein Dbh at the reaction temperature. Therefore, several synthetic, unnatural spacers were used in the activity assays. The damaged DNA template strands contained specific spacers, including alkane chains (C3, C6, and C12), polyethylene glycols (S9 and S18), and dSpacer.

Unless otherwise specified, the reaction mixtures (10 μL) contained a 0.1 μM PT DNA substrate, 200 μM single dNTP or mixed dNTPs, and a specified amount of Dbh, as shown in each figure. After incubation for the specified time at 45 °C, the reactions were stopped by adding an equal volume of loading buffer (95% formamide, 100 mM EDTA, 0.2% SDS 0.02% bromophenol blue, and 0.02% xylene cyanol). The extension products were separated by electrophoresis through a 15% polyacrylamide gel containing 8 M urea and then visualized and quantified using a Typhoon 9500 (GE Healthcare, Chicago, IL, USA).

### 2.4. Homology Modeling and Molecular Dynamics Simulation

In order to compare the effect of a spacer C3 on the TLS, PT DNA with a free or bulging spacer C3 on the template strand was first built in PYMOL-1.8 (Schrodinger LLC, New York, NY, USA) [23] and, subsequently, an optimized conformer of the PT DNA was docked into the crystal structure of Dbh (PDB ID: 3BQ1) [19] in AUTODOCK [24]. The AMBER14 suit package [25] was used to construct solvent–protein systems and perform molecular dynamics (MD) simulations. For the simulation, the protein–DNA complex and ions were solvated in a truncated octahedron box of TIP3P waters with a water thickness of 10 Å. All systems were minimized to 40,000 steps with the steepest descent method and then heated for 20 ps and equilibrated for 20 ps in the NVT ensemble at room temperature. Fifty-nanosecond MD simulation trajectories were generated for each system with ff12SB force fields. To calculate the dynamics information of each system in the converged stage, the first 20 ns of all trajectories were discarded. Furthermore, routine analysis of trajectory sampling was carried out using CPPTRAJ in Ambertools14 [26]. Based on the interactions between Dbh and the substrates, the blocking of the primer extension by spacer C3 was analyzed in terms of the distance between the 3′-OH of the primer and the α-phosphate group of the incoming dNTP.

## 3. Results

### 3.1. The Paired Primers Were More Easily Extended by Dbh on Spacer Skipping

**Figure 1:**
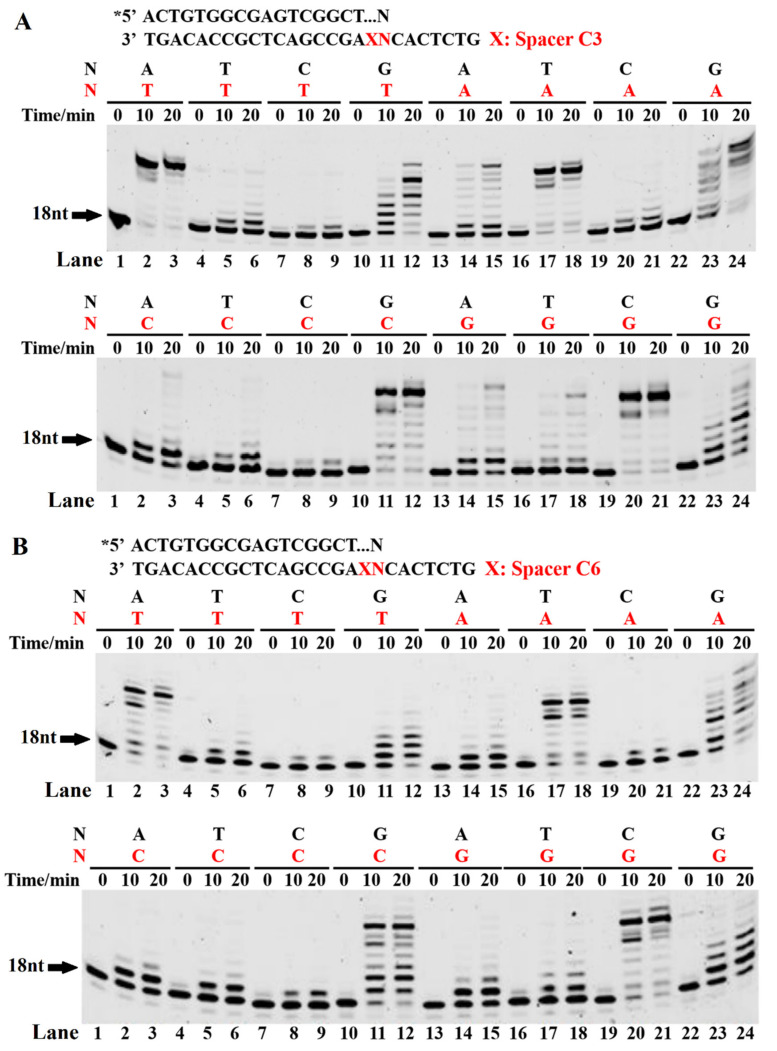
Primer extension of paired or mispaired 3′ base pairs with a bulging spacer. The PT DNA substrates are shown at the top of the figure, where the letter X denotes spacers C3 (**A**) and C6 (**B**). The first 3′ base of the primer strand is A, T, C, or G. The first base upstream of the AP site in the template strand is A, T, C, or G. Four primer strands were annealed with four different template strands to form 16 kinds of PT DNA substrates with a bulging spacer C3 or C6, which were incubated with 0.5 µM Dbh and dNTPs at 45 °C for 0, 10, 20, and 60 min. Asterisks denote the terminal fluorescent group FAM.

In order to investigate the TLS mechanism of the archaeal Y-family DNA polymerase Dbh on alkane chain spacers, we designed a set of 26-mer template strands with the spacers C3 and C6 adjacent to the different upstream 5′ bases (A/T/C/G) and a set of 18-mer 5′-FAM-labeled primer strands with different 3′ bases (A/T/C/G). The primer was extended in the presence of a dNTP. If template skipping occurred, the template primer would form various 3′ base pairs, including matched pairs (Figure 1). Our results showed that a faster and full-length extension occurred for four correctly paired 3′ base pairs of A/T or C/G (Figure 1), indicating that the Dbh mainly uses template skipping to bypass the spacers for TLS. In addition, the 3′ mismatches of G/T, G/A, and G/G also generated several products. However, the product of the G/G mismatch was probably from the extension of the paired 3′ G/C after skipping both the spacer and the adjacent G. The mismatches of G/T, G/A, G/G, T/T, T/G/, and T/C also generated a certain number of extension bands, which were likely derived from a highly error-prone synthesis, as shown for the normal DNA template (Figure S2, left panel), or from a template-independent extension activity [27]. With increasing spacer length, the skipping of the spacer and primer extension by the Dbh became more difficult (Figure 1B). These results indicated that the Dbh mainly performed TLS using a template skipping mechanism for bypassing spacers and that the correct pairing of the 3′ base pairs promoted spacer skipping and the TLS efficiency.

### 3.2. Incorporation of the First Nucleotide during Bypassing Spacer by Dbh

**Figure 2:**
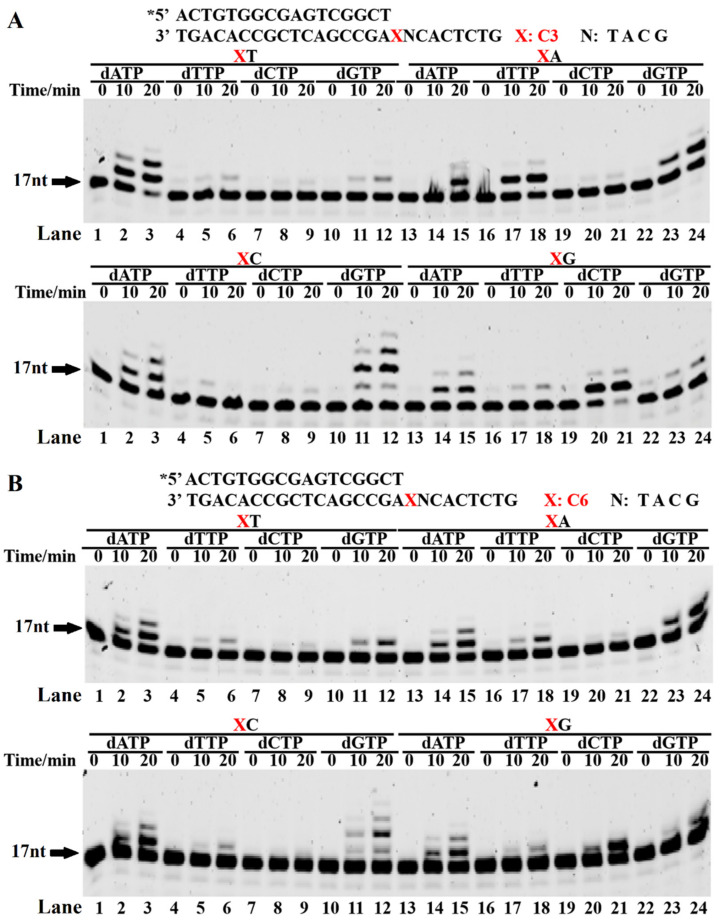
Single dNMP incorporation during AP site bypass. The PT DNA substrates are shown at the top of the figure, where the letter X denotes spacers C3 (**A**) and C6 (**B**). The first base upstream of the AP site in the template strand is A, T, C, or G. The PT DNA substrates were incubated with 0.1 µM Dbh in the presence of dATP, dCTP, dGTP or dTTP at 45 °C for 0, 10, 20, and 60 min. Asterisks denote the terminal fluorescent group FAM.

In order to further confirm that the Dbh adopted a template skipping mechanism for bypassing the spacer, the 26-mer template strands carrying a spacer C3 or C6, shown in Figure 1, were annealed with a 17-mer primer strand to place the 3′ end of the primer adjacent to the spacer (Figure 2). The primer was extended in the presence of dATP, dTTP, dCTP, or dGTP. During the incorporation of a single dNMP, the first dNMP to be incorporated matched the AP site damage or the 5′ first base of the AP site after the Dbh skipping the spacer. Therefore, whether the Dbh adopted the template skipping mechanism could be determined based on the identity of the incorporated dNMP. The extension pattern of a single dNMP (Figure 2) was very similar to the results of the above template skipping and 3′-pairing extension assay (Figure 1). When the incoming dNMP skipped the spacer and correctly paired with the next base, its incorporation occurred easily. For the XC template, more than two dGMPs were incorporated. Because two tandem C bases (X-C-C) were used as templates after skipping spacers, two dGMPs were correctly paired and incorporated. In addition to the correctly paired incoming dNMPs, some mismatched dNMPs, including A/A, G/A, A/C, and G/A, were also incorporated, with the preferential incorporation of A and G. The preferential incorporation of the mismatched dAMP and dGMP at the 3′-terminus was probably derived from the strong template-independent polymerase activity of the Dbh, which prefers dAMP and dGMP (Figure S3) [27]. With an increasing spacer length, the skipping of the spacer by the Dbh, followed by a dNTP-stabilized misalignment, became more difficult (Figure 2B).

In addition, two template strands (normal base and dSpacer, which has the most similar structure with a natural spacer) were annealed with the 17-mer primer strand to form a substrate for the incorporation of four different single dNMPs (dAMP, dTMP, dCMP, and dGMP) (Figure S2). For the normal base T template (Figure S2, left panel), more than one single dNTP was incorporated continually, which is a typical property of the low-fidelity DNA polymerase Dbh. For the dSpacer template (Figure S2, right panel), the incorporation of dAMP was consistent with the spacer C3 and C6 templates (Figure 2). The higher dGMP incorporation efficiency might have been due to the dS-T sliding together or the extension of the dGMP/T mismatch. The dTMP incorporation, comparable to that of the dGMP, might have occurred due to the X-T-C sliding together or the incorporation of the dTMP/T mismatch. The lowest incorporation efficiency of the dCMP might have been due to the incorporation of the dCMP/dS or dCMP/T mismatch. Another interpretation of the differences in the unpaired dNMP incorporation efficiencies was that the template-independent polymerase activity preferred dAMP and dGMP (Figure S2) [27]. Based on the above results, we concluded that the Dbh mainly used template skipping accompanied by a dNTP-stabilized misalignment to bypass the AP site analogs, resulting in a spacer-deletion extension.

### 3.3. Incorporation of the First Nucleotide Downstream of AP Sites Was the Most Difficult

**Figure 3:**
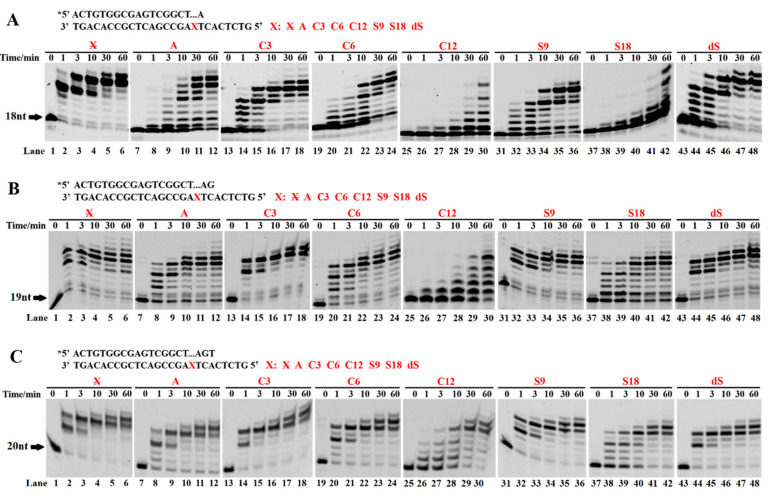
Primer extension with increasing pairing numbers of 3′ base pairs with a bulging spacer. The PT DNA substrates are shown at the top of the figure. The letter X denotes a skipped base A and spacers C3, C6, C12, S9, S18, and dS. The symbol X denotes the normal DNA template. The PT DNAs, with 3′ base pairs of 1 bp (**A**), 2 bp, (**B**) and 3 bp (**C**) after the bulging spacer or base A, were used as substrates to characterize the extension reactions. The PT DNA substrates were incubated with 0.1 µM Dbh and dNTPs at 45 °C for 0, 1, 3, 10, 30, and 60 min. Asterisks denote the terminal fluorescent group FAM.

To investigate the rate-limiting step during TLS by the Dbh, we designed PT DNA substrates with a 1-mer loop of A, C3, C6, C12, S9, S18, and dSpacer. Three groups of PT DNA substrates with 3′-pairing regions 1 bp, 2 bp, and 3 bp downstream of the 1-mer loop were used for the TLS assays and compared with the normal template. The results showed that, compared with the normal template, the spacer greatly decreased the TLS efficiency in the order 1 bp < 2 bp < 3 bp (Figure 3). Moreover, the TLS efficiency decreased with the increasing molecular length of the spacer. These results suggested that the correct base pairing +1/2 downstream of the AP site facilitated the efficiency of the elongation of the primer end by the Dbh. Furthermore, the TLS of the 1-mer spacer loop was compared with that of the spacer–base mismatch (Figure S4). For three base pairs of 1 bp, 2 bp, and 3 bp, the TLS efficiency of the 1-mer loop was largely higher than that of the spacer–base mismatch, especially for the 1 bp base pair. These results further confirmed that the Dbh mainly bypassed the AP site via spacer-skipping accompanied by a dNTP-stabilized misalignment mechanism.

**Figure 4:**
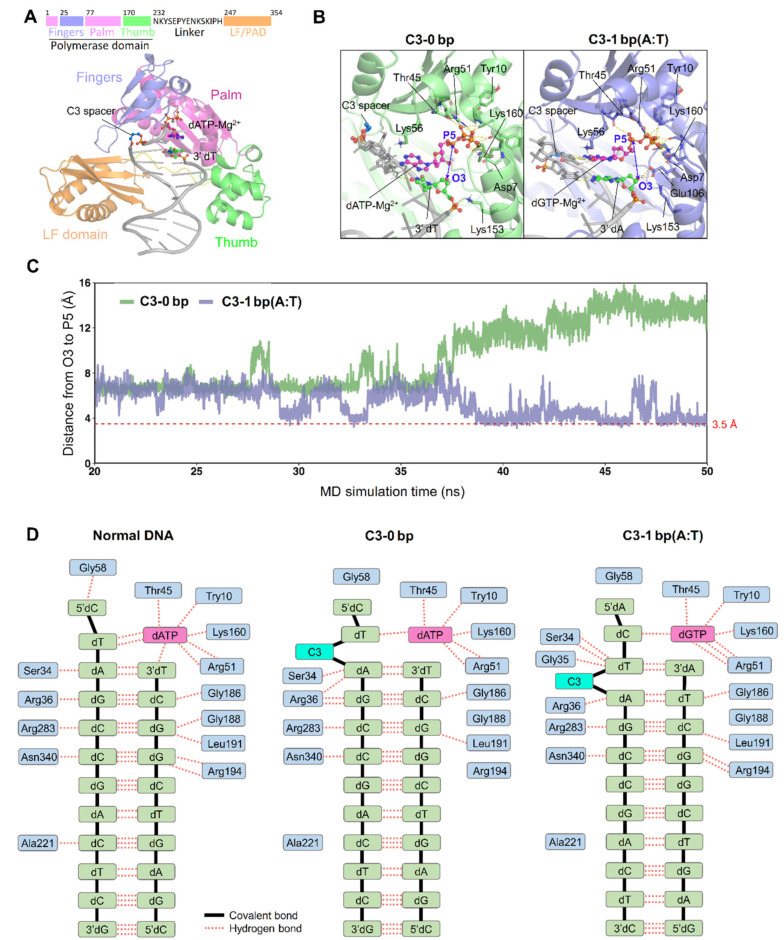
Effect of spacer C3 on TLS efficiency. (**A**) Schematic depiction of the domain composition of Dbh and its modeled structure with bound PT DNA carrying a free spacer C3. (**B**) Modeling of the formation of the phosphodiester bond between the incoming dNTP and the 3′-OH of the primer paired with the DNA template strand with a free (left panel) or bulging (right panel) spacer C3. (**C**) Distance between the 3′-OH of the primer strand and the alpha phosphate group of the incoming dNTP. PT DNA with a free (C3-0 bp) or bulging (C3-1 bp) spacer C3 was used to calculate the distance. (**D**) Hydrogen bond interactions between Dbh and normal PT DNA substrate (left panel) or the counterparts carrying a spacer C3 in the DNA template strand (the middle panel is for free spacer C3, and the right panel is for bulging spacer C3).

The modeled complex structure of the Dbh, PT DNA, and the incoming dNTP (Figure 4A,B) also showed that the formation of a phosphodiester bond was favored for the PT with the bulging spacer C3 (forming a 1 bp paired 3′-terminus upon sliding the spacer; Figure 4B, right panel) compared with that with the free spacer C3 (without sliding the spacer to form a 1 bp paired 3′-terminus; Figure 4B, left panel). The distance between the incoming dATP and the 3′-OH group of the primer strand also indicated the favorable formation of the new phosphodiester bond after sliding the spacer and forming the 1 bp paired 3′ primer terminus (Figure 4C, C3-1 bp vs. C3-0 bp). Compared with the normal PT DNA substrate, the insertion of the spacer C3 caused significant fluctuations at the residues 34S, 35G, 36R, G58, G118, G160, G186, G188, L191, 194R, A221, R283, and N340, which weakened the hydrogen bond interactions between the DNA and Dbh and blocked the formation of new phosphodiester bonds (Figure 4D).

### 3.4. Effect of the Structure of Sliding PT DNA on the TLS Efficiency of Dbh

**Figure 5:**
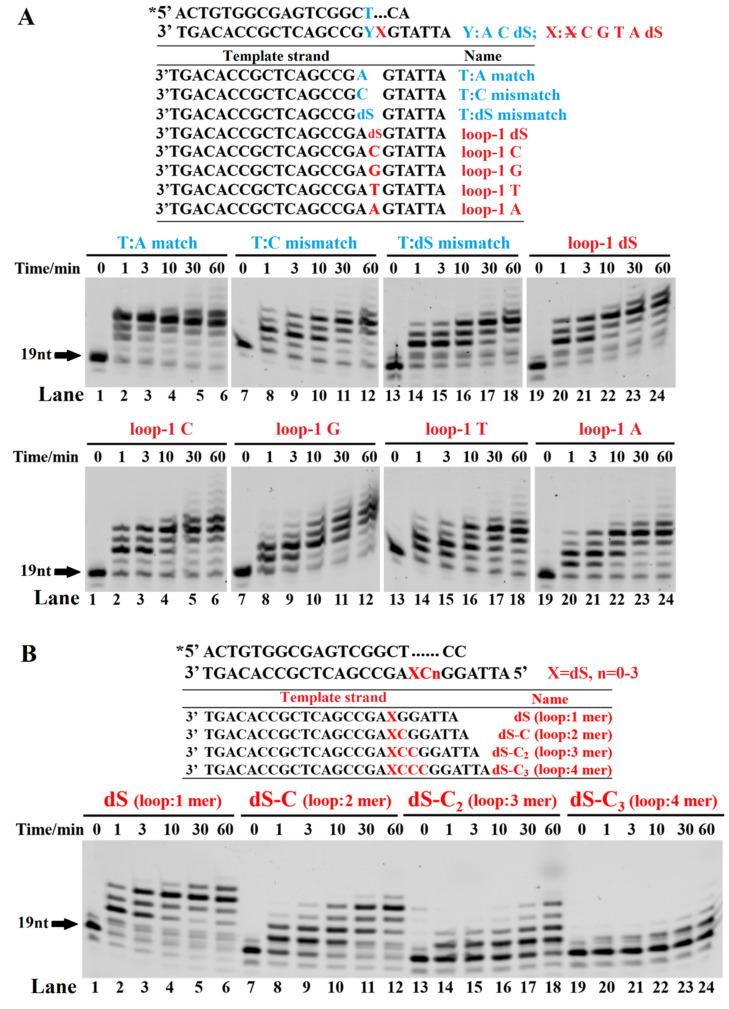
Effect of the bulging loop on TLS. The PT DNA substrates are shown at the top of the figure, where the letter X denotes bases A, T, C, G, and dSpacer, and the letter Y denotes bases A, C, and dSpacer. The symbol X denotes without spacer or base. (**A**) Effect of the base mismatches (T:C mismatch and T:dS mismatch) and a bulging base loop or dSpacer loop (1-mer loops of dS, C, G, T, and A) on TLS efficiency. (**B**) Effect of the size of the bulging loop (dS, dS-C, dS-C_2_, and dS-C_3_) on TLS efficiency. The PT DNA substrates were incubated with 0.1 µM Dbh and dNTPs at 45 °C for 0, 1, 3, 10, 30, and 60 min. Asterisks denote the terminal fluorescent group FAM.

To investigate the effect of the structure of the sliding loop in the DNA template strand on the TLS efficiency of the Dbh, we designed five 1-mer sliding loops with different compositions of C, G, T, A, and dSpacer, two mismatches of T:C and G:dS, and one T:A match as a positive control (Figure 5A). The mismatches of T:C and G:dS were extended less efficiently than the corresponding 1-mer loops of loop-1 C and loop-1 dS, indicating that the spacer skipping mechanism is mainly used by the Dbh. The TLS efficiency of the four 1-mer loops of the bases was in the order loop-1 C > T ≈ A ≈ G, and loop-1 C exhibited an extension percentage comparable to that of loop-1 dS (Figure 5A). Furthermore, the effect of the size of the bulging loop on the TLS efficiency of the dSpacer was characterized. With the increase in the number of the skipped dSpacers and C bases, the bulging loops became larger, the corresponding TLS efficiency gradually decreased, and the loop of dSC_3_ almost completely inhibited the primer extension (Figure 5B). In summary, the size of the skipped loop in the DNA template strand had a greater effect on the TLS efficiency of the Dbh, while the base composition of the skipped loop of the same size had a very slight effect.

### 3.5. Key Amino Acids of Dbh Involved in Stabilizing the Bulging Damaged Base

**Figure 6:**
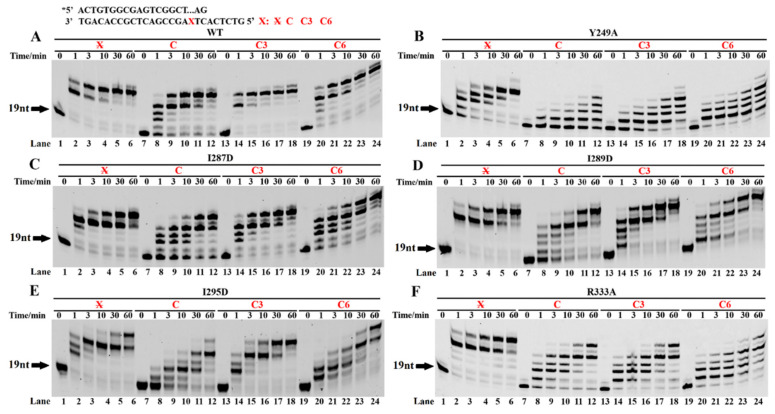
TLS by LF domain mutants of Dbh. The PT DNA substrates are shown at the top of the figure, where the letter X denotes base C and spacers C3 and C6. The symbol X denotes the normal DNA template. The PT DNA substrates, with 3′-pairing two normally paired base pairs downstream of the AP site in the template strand, were incubated with dNTPs and 0.2 µM wt Dbh (**A**), or the mutant Y249A (**B**), I287D (**C**), I289D (**D**), I295D (**E**), or R333A (**F**) at 45 °C for 0, 10, 20, and 60 min. 2 bp downstream of the 1-mer loop. Asterisks denote the terminal fluorescent group FAM.

The crystal structure of the Dbh shows that the LF domain determines the TLS specificity [19]. Based on the schematic diagram of the interactions between the Dbh and the DNA substrate [19], it was found that the amino acid residues Y249, I287, I289, I295, and R333 in the LF domain of the Dbh interact with the DNA damage sites, including the AP site analogs. The PT DNA substrates, with a 2-bp paired 3′ terminus and a 1-mer loop structure (Figure 6, panels C, C3, and C6) or normal template (Figure 6, panel X), were used to characterize the potential function of the above-conserved residues. Considering that those mutations also affected the efficiency of DNA elongation on the normal template, the extension product ratio of the damaged to the normal template was used to confirm the specific function of mutations on TLS. The extension product ratios of the Y249A, I295D, and R333A mutants were significantly lower than those of the wild-type Dbh (Figure 6B,E,F), but the ratios of the I287D and I289D mutants (Figure 6C,D) did not change much compared with those of the wild-type Dbh, indicating that the amino acid residues Y249, R333, and I295 in the LF domain of the Dbh might play a major role in the TLS process.

### 3.6. Effect of Residues Interacting with Normal DNA Strands on TLS

**Figure 7:**
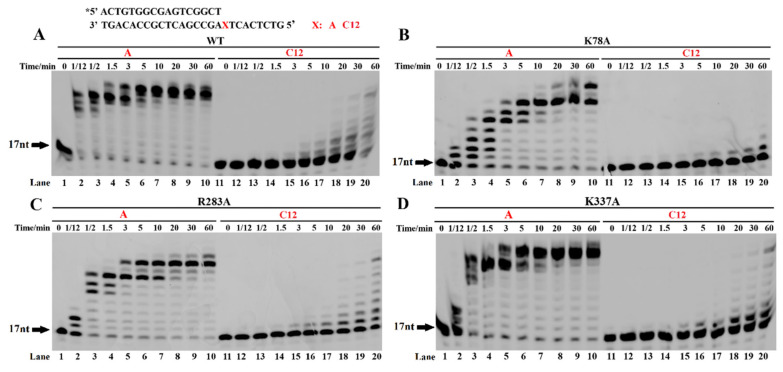
TLS by palm and linker mutants of Dbh. The PT DNA substrates are shown at the top of the figure, where the letter X denotes base A (normal DNA template) and spacer C12. DNA substrates were incubated with dNTPs and 0.2 µM wt Dbh (**A**), or the mutant K78A (**B**), R283A (**C**), or K337A (**D**), at 45 °C for 0, 10, 20, and 60 min. Asterisks denote the terminal fluorescent group FAM.

The main residues interacting with the normal DNA strands also participate in TLS [19]. Based on the crystal structure of the Dbh and DNA, these residues mainly include K78, R283, and K337. K78 is located at the N-terminus of the palm subdomain of the catalytic core domain; R283 and K337 are located in the LF domain. The effects of the K78, R283, and K337 residues on DNA synthesis were compared using the normal and the damaged templates. The percentages of their extended substrates were in the following order: K78A < R283A ≈ K337A < wt Dbh for the spacer C12 template, with the incorporation of only 1–2 nucleotides into the primer for K78A (Figure 7). However, the order for the normal DNA template was as follows: K78A < R283A < K337A ≈ wt Dbh K337A (Figure 7). Moreover, the truncation of the LF domain decreased the DNA synthesis’ activities for both the normal and the damaged DNA templates, with the incorporation of only one nucleotide into the primer (Figure S5). These results suggested that K78 and R283 in the palm subdomain of the catalytic core domain played a role in both normal synthesis and TLS, and the LF domain, especially K337, played a major role in TLS.

## 4. Discussion

Here, we found that the Dbh efficiently carried out TLS across the dS/C3/C6 spacer, which mimics an abasic site. However, a previous report showed that the Dbh could not bypass an abasic lesion [15]. The discrepancies in the results were likely derived from the different experimental conditions used during the activity assay. In our assay, more Dbh was used, and the ratio of the Dbh to the PT DNA substrate was five. Our results further suggested that the Dbh might use template skipping accompanied by a dNTP-stabilized misalignment to bypass the spacer damage, resulting in a single-base deletion. The incorporation of the first nucleotide downstream of the damage site was the major rate-limiting step during the TLS of the AP sites; in other words, it was the most difficult and slow step. The Dbh efficiently extends primers with normally paired 3′ base pairs of A/T or C/G downstream of the bulging spacer (Figure 1), suggesting that the correct pairing of base pairs downstream of the spacer promotes template sliding of the AP site and improves the Dbh TLS efficiency. In addition, because the Dbh is an error-prone DNA polymerase, more mismatches are generated during the TLS of AP sites (Figure 1).

The mechanism of the template spacer skipping accompanied by a dNTP-stabilized misalignment was further confirmed by the easy and fast incorporation of a single dNMP, which, correctly paired with the 5′ first base of the AP site after the Dbh, skipped the spacer on the template strand (Figure 2). In contrast, if the dNTP did not pair with the 5′ first base of the skipped spacer on the template strand, it was hardly incorporated or was incorporated at a very slow rate. These results imply that the Dbh mainly uses spacer sliding accompanied by a dNTP-stabilized misalignment to incorporate the first nucleotide after easily skipping the AP site analogs. During the incorporation of a single dNMP, an AMP extension with similar efficiency was observed for the unpaired template bases of C, G, and A (Figure 2). This result could have been derived from the template-independent extension activity of the Dbh, i.e., the A-rule, where the AMP is preferably incorporated at the primer 3′-OH, independent of the DNA template (Figure S3). The A-rule or misincorporation/misalignment mechanism is adopted by the Dbh when a larger spacer exists, or the 5′ base of the AP site does not correctly pair with the incoming dNTP. Moreover, our results showed that correct base pairing +1/2/3 downstream of the AP site facilitated the efficiency of the primer elongation by the Dbh. This result is similar to the extension of a single-base deletion by the Dbh [19], where for the different locations of the jumping base upstream of the nascent base pair, the Dbh extends the single-base deletion primer in the speed order +3 > +2 >> +1 [19].

The Y-family DNA polymerases all consist of an N-terminal polymerase catalytic core domain and a C-terminal LF domain/PAD that helps bind DNA [13,14,15,28,29]. Interestingly, the amino acid sequence of the polymerase catalytic core domain is highly conserved, while the sequence similarity of the LF domain/PAD is very low. Thus, it is likely that the LF domain/PAD is the main determinant of the reaction properties of TLS. The Dbh is highly prone to single-base deletion because the gap between the polymerase catalytic core domain and C-terminal LF domain of the Dbh provides ample space to accommodate the skipped base [15,19]. The Dbh does not strictly limit the entry of the mismatched template base–dNTP pair into the enzymatic active center as the high-fidelity replicative polymerases have [30]. This lower stringency may allow template misalignment to occur more easily [19], and the low sustained synthesis capacity of the Dbh provides more opportunities for the DNA strand rearrangement [29,31]. Our results also indicate that the skipping of the spacer is the first step and the correct pairing of incoming dNTPs, where the 5′ first base of the skipped spacer is the second step for the efficient TLS of the AP site analogs by the Dbh.

The spatial orientation of the C-terminal LF domain relative to the catalytic core domain of the polymerase may be a key factor in determining the molecular mechanism of a single-base or spacer deletion employed by the Dbh. The three conserved amino acid residues (Y249, R333, and I295) on the LF domain play a key role in the TLS of the spacers. In addition to the LF domain, the residues for binding DNA are also important for TLS; K78, R283, and K337 all interact with the DNA strand [19]. We tested the importance of the amino acid residues contacting the template bases. Residues K78 and R283 are required for normal DNA synthesis and the TLS of the spacer. In contrast to the mutants K78A and R283A, the truncation of the C-terminal LF domain or the mutation K337A led to a greater decrease in the extension for TLS than normal DNA synthesis (Figure 7 and Figure S5), suggesting that they play a specific, major role in TLS.

In summary, the Dbh mainly uses template skipping accompanied by a dNTP-stabilized misalignment to bypass the AP site analogs, and the incorporation of the first nucleotide across the AP site is the main rate-limiting step. The conserved residues Y249, R333, I295, and K337A in the LF domain play key roles during TLS by the Dbh.

## Data Availability

Not applicable.

## Supplementary Materials

The following are available online at https://www.mdpi.com/article/10.3390/ijms23052729/s1.
