# Supplementary material for: Mechanism Underlying the Bypass of Apurinic/Pyrimidinic Site Analogs by Sulfolobus acidocaldarius DNA Polymerase IV
Source: Int J Mol Sci. 2022 Mar 1;23(5):2729. doi: 10.3390/ijms23052729 (PMC8910976; doi:10.3390/ijms23052729)
Supplement: Supplementary file 1 [file ijms-23-02729-s001.zip › ijms-1581961-supplementary.pdf]

Supplementary materials

Supplementary Figures

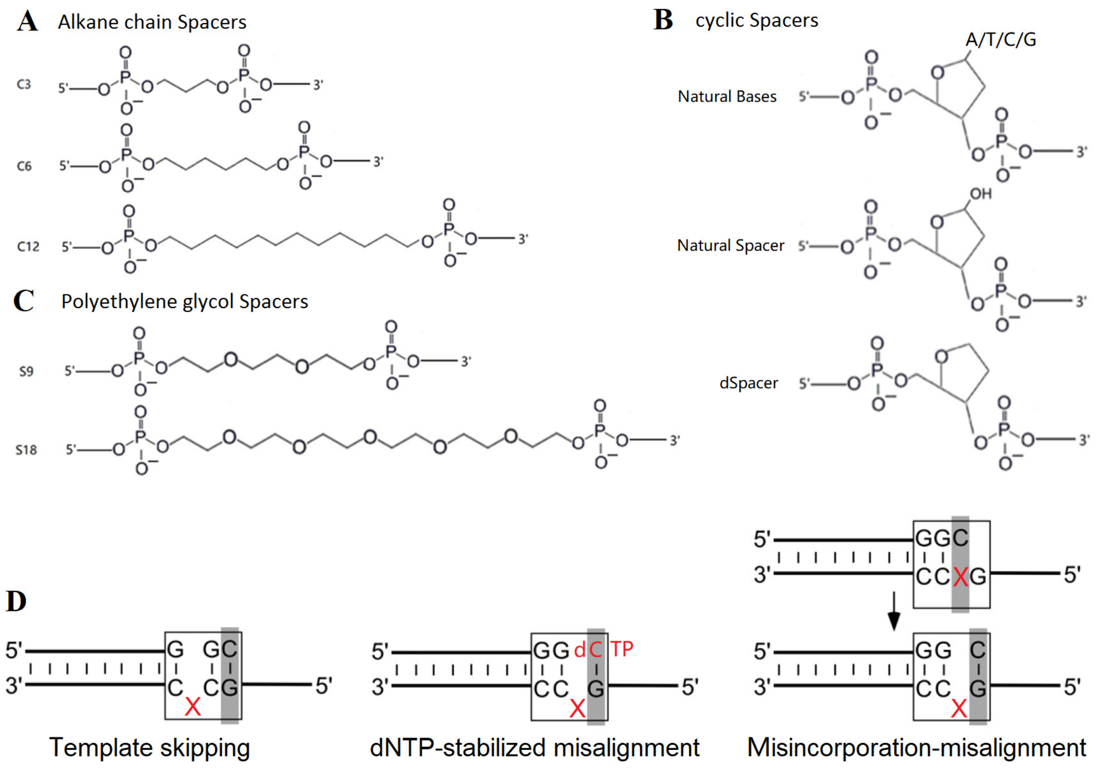

Figure S1 Structures of various AP site analogs and molecular mechanisms of translesion synthesis. The molecular structure of (**A**) alkane chain spacers (Spacer Cn), (**B**) cyclic spacers (natural spacer, and synthetic dSpacer and normal bases), (**C**) polyethylene glycol spacers (Spacer 9 and Spacer 18), and (**D**) three mechanisms of translesion synthesis of spacers, which are denoted by red letter X.

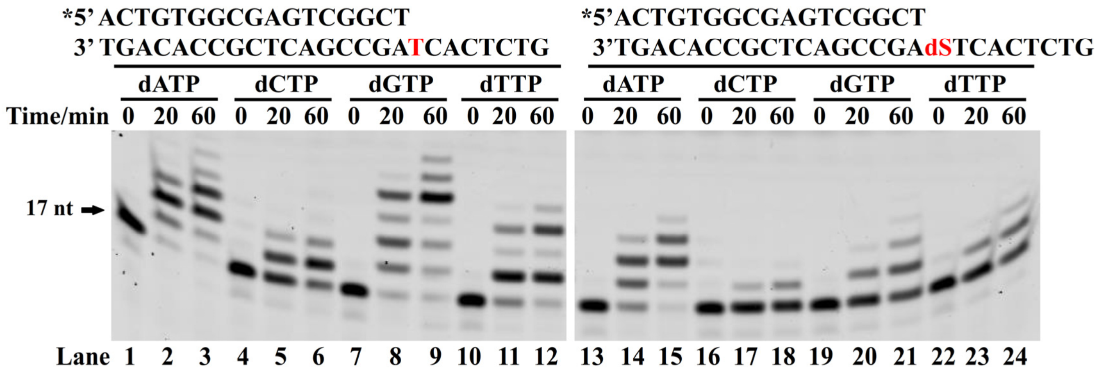

Figure S2 Single dNTP incorporation opposite to the normal base and dSpacer. The PT DNA substrates are shown at the top of the figure. The first base downstream of dSpacer in the template strand is T. The PT DNA substrates were incubated with 0.1 μM Dbh in the presence of dATP, dCTP, dGTP or dTTP at 45°C for 0, 10, 20, and 60 min.

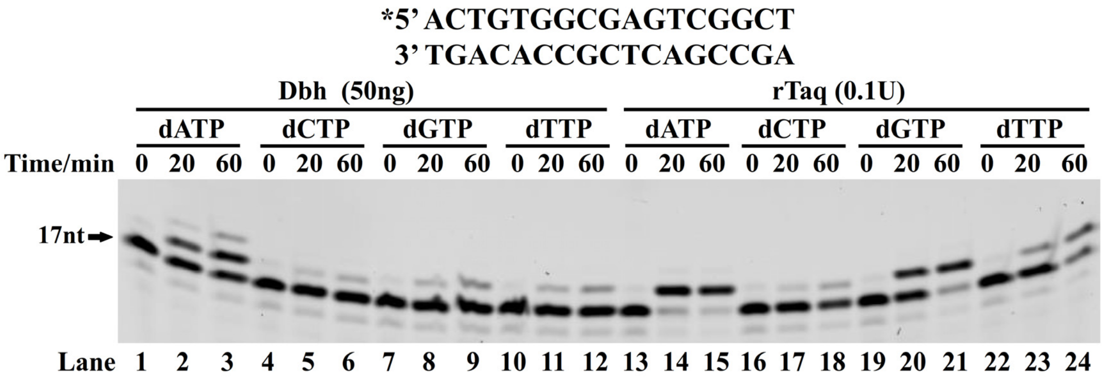

Figure S3 Template-independent extension activity of Dbh and Taq DNA polymerase. The blunt double-stranded DNA substrate, shown at the top of the figure, was incubated with 0.1 μM Dbh or 0.1 U recombinant Taq DNA polymerase at 45°C for 0, 20 and 60 min in the presence of a single dNTP.

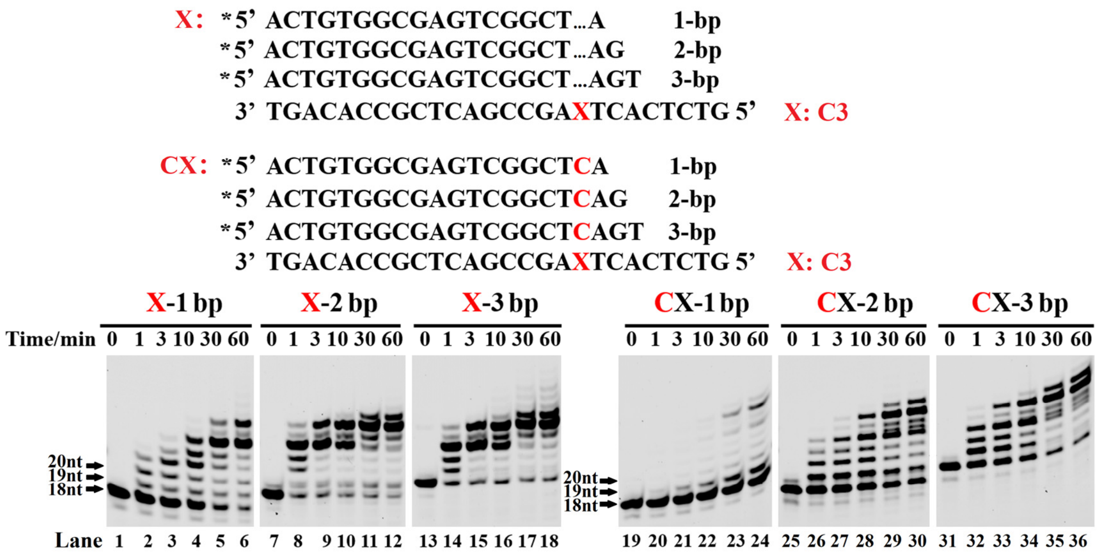

Figure S4 Primer extension with increasing pairing numbers of 3' base pairs with bulging or mismatched spacer C3. The PT DNA substrates are shown at the top of the figure, where the letter X denotes spacer C3. X-1 bp, X-2 bp and X-3 bp denote the PT DNA substrates with the 3' base pairs 1 bp, 2 bp, and 3 bp after the bulging spacer C3, respectively. CX-1 bp, CX-2 bp and CX-3 bp denote the PT DNA substrates with the 3' base pairs 1 bp, 2 bp, and 3 bp after the mismatch of base C and spacer C3, respectively. The PT DNA substrates were incubated with 0.1 μM Dbh and dNTPs at 45°C for 0, 1, 3, 10, 30 and 60 min.

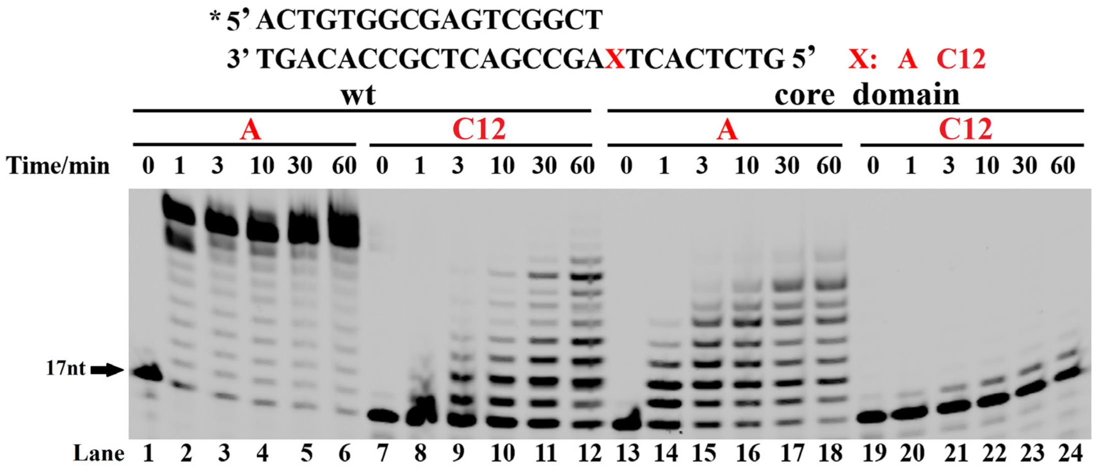

Figure S5 Comparison of the polymerase activities of wt Dbh and its core domain. The PT DNA substrates are shown at the top of the figure, where the letter X denotes base A and spacer C12. The PT DNA substrates were incubated with 0.5 μM wt Dbh or the core domain and dNTPs at 45°C for 0, 10, 20, and 60 min.

Supplementary Table

Table S1 The sequences of primers for constructing site-directed mutated Dbh expression vectors

Primer	Sequence (5'-3') [a]
K78A-F	GCCTATGAGAgcACCGATTTATGAGGCATTCTCA
K78A-R	CATAAATCGGTgcTCTCATAGGCACATATATTGCG
Y249A-F	TCATGGAAGAgcTTTAACTTTACCCTATAACACAA
Y249A-R	TAAAGTTAAAgcTCTTCCATGAGGAATTTTACTT
R283A-F	TATTCCAATGgcAATAACTGTTATAGCTATTATGG
R283A-R	AACAGTTATTgcCATTGGAATACCATTAACCTTA
I287D-F	ATAACTGTTgatGCTATTATGGAAGATCTAGAT
I287D-R	CCATAATAGCatcAACAGTTATTCTCATTGGAATA
I289D-F	TGTTATAGCTgaTATGGAAGATCTAGATATTCTA
I289D-R	ATCTTCCATAtcAGCTATAACAGTTATTCTCATT
I295D-F	AGATCTAGATgaTCTAAGTAAGGGAAAAAGTTTAAG
I295D-R	CTTACTTAGAtcATCTAGATCTTCCATAATAGCT
R333A-F	AAATGTACGAgcAATAGGAGTAAAATTAGATAACATA
R333A-R	TACTCCTATTgcTCGTACATTTCTTCTTTTATCTC
K337A-F	AATAGGAGTAgcATTAGATAACATAATAATCAATAAGA
K337A-R	GTTATCTAATgcTACTCCTATTCTTCGTACATTT

[a] The red bases denote the site-mutated bases.
